# Diagnostic Accuracy of Individual and Multimodal Pulp Sensibility Tests Compared With Radiographic Assessment for the Diagnosis of Pulpal Health: A Prospective Diagnostic Accuracy Study

**DOI:** 10.7759/cureus.113914

**Published:** 2026-08-03

**Authors:** N. Tulasi Priya, Amulya Vanapatla, Chaitanya Puppala, Aishwarya T N, Naresh Gaddala, Naseer Mohammad

**Affiliations:** 1 Department of Conservative Dentistry and Endodontics, Government Dental College and Hospital, Hyderabad, IND; 2 Department of Conservative Dentistry and Endodontics, Mamata Dental College, Khammam, IND; 3 Department of Conservative Dentistry and Endodontics, Sri Sai College of Dental Surgery and Hospital, Vikarabad, IND; 4 Department of Oral and Maxillofacial Surgery, Sri Sai College of Dental Surgery and Hospital, Vikarabad, IND

**Keywords:** dental pulp necrosis, pulp test, pulse oximetry, roc curve, sensitivity and specificity

## Abstract

Introduction: The diagnostic performance of individual pulp sensibility tests and multimodal diagnostic strategies for differentiating vital and necrotic pulps in teeth with endodontic-periodontal lesions remains uncertain. Therefore, this prospective diagnostic accuracy study evaluated and compared the diagnostic performance of individual pulp sensibility tests and multimodal diagnostic models with a composite reference standard.

Materials and methods: Ninety-eight patients aged 18-65 years with endodontic-periodontal lesions underwent cold testing, heat testing, electric pulp testing, and pulse oximetry. The composite reference standard comprised radiographic assessment and intraoperative bleeding during the preparation of the access cavity. Diagnostic accuracy parameters such as sensitivity, specificity, predictive value, and area under the curve (AUC) were calculated for each test. Three multimodal models (parallel "OR," serial "AND," and weighted logistic regression) were developed and compared with the best-performing individual test using DeLong's test and McNemar's test.

Results: A total of 98 patients were included in the study, comprising 52 (53.1%) males and 46 (46.9%) females, with 175 teeth evaluated. Pulse oximetry demonstrated the highest individual diagnostic accuracy (AUC = 0.905, sensitivity = 93.0%, specificity = 88.0%), followed by the electric pulp tester (AUC = 0.860), cold test (AUC = 0.825), and heat test (AUC = 0.750). The parallel model achieved the highest sensitivity (97.0%), whereas the serial model achieved the highest specificity (97.3%). The weighted logistic regression model provided the best overall performance (AUC = 0.933, sensitivity = 91.0%, specificity = 90.7%), significantly outperforming pulse oximetry (p = 0.032) and the parallel model (p < 0.001). Pulse oximetry emerged as the strongest independent predictor of pulp necrosis in multivariable analysis (adjusted OR = 11.13, p < 0.001).

Conclusion: Pulse oximetry demonstrated the highest diagnostic accuracy among the individual tests evaluated for assessing pulpal health. Although the parallel and serial multimodal models achieved the highest sensitivity and specificity, respectively, the weighted logistic regression model showed the best overall diagnostic performance by integrating information from multiple tests. These findings suggest that a multimodal approach incorporating pulse oximetry, electric pulp testing, and thermal testing may improve diagnostic accuracy and facilitate more informed treatment planning in endodontic practice.

## Introduction

An accurate assessment of pulp vitality is fundamental for establishing an appropriate endodontic diagnosis and selecting the most suitable treatment strategy. Differentiating between vital and necrotic pulp is particularly challenging in teeth presenting with endodontic-periodontal lesions, where clinical signs and symptoms often overlap and conventional diagnostic methods may provide inconsistent findings [1]. Although periapical radiographic examination remains an indispensable component of endodontic diagnosis because it reveals periapical pathosis and periodontal ligament alterations, radiographic changes generally represent the consequences of pulpal disease rather than the actual physiological status of the pulp [2].

Consequently, reliance on radiographic findings alone may result in delayed or inaccurate diagnosis, particularly during the early stages of pulpal degeneration [2]. Conventional pulp sensibility testing, including the cold test, heat test, and electric pulp tester, evaluates the sensory response of neural tissue rather than the vascular integrity of the pulp, making them susceptible to false-positive and false-negative results in cases involving trauma, calcified canals, immature teeth, or recently restored teeth [1,3]. In contrast, pulse oximetry has emerged as a promising non-invasive diagnostic modality because it measures pulpal blood oxygen saturation, thereby providing a more direct assessment of pulp vitality independent of neural response [4].

Despite the demonstrated advantages of individual diagnostic tests, none have consistently achieved ideal sensitivity and specificity across diverse clinical situations [1]. Since clinicians routinely integrate information from multiple diagnostic modalities rather than relying on a single test, combining different pulp vitality tests into multimodal diagnostic models may improve the overall diagnostic performance by balancing the strengths and limitations of individual methods. However, evidence comparing multimodal diagnostic strategies to conventional single-test approaches remains limited. Therefore, the present prospective diagnostic accuracy study was designed to evaluate and compare the diagnostic performance of individual pulp sensibility tests, including the cold test, heat test, electric pulp tester, and pulse oximetry, with radiographic assessment as the reference standard and to determine whether multimodal diagnostic models provide superior diagnostic accuracy for identifying vital and necrotic pulps. The objectives of this study were to compare the sensitivity, specificity, positive predictive value (PPV), negative predictive value (NPV), receiver operating characteristic (ROC) curves, and area under the curve (AUC) of individual pulp sensibility tests; to assess the diagnostic performance of parallel, serial, and weighted logistic regression multimodal models; and to identify the most accurate diagnostic approach for routine clinical assessment of pulpal health.

## Materials and methods

This prospective diagnostic accuracy study was conducted in the Department of Conservative Dentistry and Endodontics, Sri Sai College of Dental Surgery and Hospital, Vikarabad, India, over a period of six months from May 2023 to April 2024. The study protocol was reviewed and approved by the Institutional Ethics Committee (37-SSCDS/Acad/Ethical/2023) of the college before participant recruitment, and all procedures were performed in accordance with the ethical principles outlined in the Declaration of Helsinki. Written informed consent was obtained from all participants prior to enrollment.

Participants were recruited using a consecutive sampling strategy. Every eligible patient presenting during the study period was screened against the predefined inclusion and exclusion criteria, and those who met the eligibility criteria and provided written informed consent were enrolled consecutively until the target sample size was reached. This non-probability sampling approach minimized investigator selection bias and ensured that the study population reflected the spectrum of patients encountered in routine clinical practice. The patients aged 18-65 years presenting with endodontic-periodontal lesions involving the maxillary and mandibular anterior teeth and premolars and requiring pulp vitality assessment prior to endodontic or periodontal treatment were recruited. The age range of 18-65 years was selected to include the adult population most commonly encountered in clinical practice while minimizing the influence of developmental factors associated with immature teeth. Although age-related pulpal changes may occur in older adults, participants within this range were considered representative of the target clinical population. A summary of the eligibility criteria is presented in Table 1.

**Table 1:** Inclusion and exclusion criteria for study participants.

Inclusion criteria	Exclusion criteria
Mature permanent teeth (completely formed apices) with a clinical indication for pulpal health evaluation based on presenting symptoms (e.g., pain, sensitivity) or routine radiographic findings.	Teeth with incomplete root formation (open/immature apices) or active internal/external root resorption.
Teeth exhibiting radiographic evidence suggestive of pulpal necrosis, including: periapical radiolucency, widening of the periodontal ligament (PDL) space, to enable a valid diagnostic accuracy assessment.	Teeth with full-coverage crowns, extensive multi-surface restorations, deep carious lesions, or orthodontic bands/appliances that physically prevent accurate placement of sensibility test stimuli (cold, heat, or electric pulp testing).
Teeth with sufficient coronal structure and accessible pulp chamber space to permit standardized administration of all individual and multimodal sensibility tests without test-retest interference.	Teeth with a documented history of root fracture, calcific metamorphosis (canal obliteration), or any prior endodontic treatment (access opening, instrumentation, or medication).
Patients aged 18-65 years who are willing to undergo sequential pulp sensibility testing (cold, heat, electric, and multimodal combinations) alongside periapical/panoramic radiographic assessment as per the prospective study protocol.	Patients currently taking centrally acting analgesics, sedatives, or neuroleptic medications that alter pain perception, or those with cognitive/neurological conditions that preclude reliable subjective response to sensibility testing.

Sample size estimation was performed using G*Power software version 3.1.9.2 (Heinrich Heine University, Düsseldorf, Germany) based on an anticipated prevalence of approximately 10% of non-vital teeth reported in a previous study. Assuming a two-sided 95% confidence interval, 80% statistical power, 5% level of significance, and 5% margin of error, the minimum required sample size was calculated as 140 teeth [5]. To compensate for possible exclusions and incomplete data, an additional 25% were incorporated, resulting in a final sample of 175 teeth.

The final pulpal diagnosis was established using a composite reference standard comprising radiographic assessments and intraoperative clinical findings. Standardized digital periapical radiographs were obtained using the paralleling technique and were independently evaluated by two blinded endodontists (NTP and AV). Radiographic evidence of periapical radiolucency measuring at least 1 mm or periodontal ligament widening greater than twice the normal width, together with the absence of pulpal bleeding upon access cavity preparation and canal negotiation, was considered to confirm pulpal necrosis. Each eligible tooth subsequently underwent four pulp sensibility tests performed by a single calibrated examiner (GN) in a randomized sequence with a five-minute interval between tests to prevent sensitization.

The cold test was performed using Endo-Ice® Refrigerant Spray (Coltene/Whaledent Inc., Cuyahoga Falls, OH, USA) applied to a cotton pellet and placed on the middle third of the buccal surface for five seconds, with any painful or tingling sensation recorded as a positive response. The heat test was performed using heated gutta-percha cones (Dentsply Sirona Endodontics, Ballaigues, Switzerland) applied to the lubricated buccal surface, and the patient's response was recorded as either present or absent. The electric pulp test was performed using the Digitest® Electric Pulp Tester (Parkell Inc., Brentwood, NY, USA) with a conducting medium placed on the incisal or cuspal surface, and a response elicited before the maximum output of 80 units was considered indicative of vitality. Pulse oximetry was performed using a Contec CMS60D Pulse Oximeter (Contec Medical Systems Co., Ltd., Qinhuangdao, Hebei, China) equipped with a customized dental sensor clip positioned on the tooth crown. Teeth demonstrating a stable pulse waveform with oxygen saturation exceeding 70% were classified as vital, whereas the absence of a detectable waveform or oxygen saturation reading indicated pulpal necrosis.

To enhance the measurement reliability, all diagnostic procedures were standardized before the commencement of data collection. The principal investigator performed calibration under the supervision of an experienced endodontist by evaluating 20 teeth that were not included in the final study sample. The assessments were repeated after a two-week interval to determine intra-examiner reproducibility. Agreement between repeated observations was evaluated using Cohen's kappa coefficient, which yielded excellent intra-examiner agreement (κ = 0.91). Similarly, the agreement between the two blinded endodontists interpreting the periapical radiographs demonstrated excellent inter-examiner reliability (κ = 0.89). 

To minimize observer bias, all four pulp sensibility tests (cold test, heat test, electric pulp test, and pulse oximetry) were performed independently by a single calibrated examiner (NG) in a randomized sequence with a five-minute interval between tests. The result of each test was recorded immediately before performing the subsequent test, and the findings of one test were not used to influence the interpretation of another. The examiner performing the clinical tests was blinded to the radiographic findings, while the two endodontists (NTP and AV) who independently interpreted the radiographs were blinded to the clinical test results. The final reference diagnosis was established only after completion of all diagnostic procedures and integration of the predefined reference standard, thereby minimizing observer and interpretation bias.

Statistical analysis was performed using IBM SPSS Statistics for Windows, version 25.0 (IBM Corp., Armonk, NY, USA) and R software version 4.3.1 (R Foundation for Statistical Computing, Vienna, Austria). Data normality was assessed using the Shapiro-Wilk test. Continuous variables are expressed as mean ± standard deviation, whereas categorical variables are presented as frequencies and percentages. Diagnostic accuracy parameters including sensitivity, specificity, PPV, NPV, Youden's index, ROC curves, and AUC with 95% confidence intervals were calculated for each individual pulp sensibility test. Three multimodal diagnostic models, namely the parallel ("OR") model, serial ("AND") model, and weighted generalized equation estimate (GEE) logistic regression model, were subsequently developed and compared with the best-performing individual test. Predicted probabilities for the weighted model were generated using binary logistic regression, and the optimal cut-off value was determined using Youden's index. Differences in AUCs between diagnostic models were analyzed using DeLong's test, whereas paired sensitivities and specificities were compared using McNemar's test. Statistical significance was set at p < 0.05.

## Results

A total of 98 patients were included in the study, comprising 52 (53.1%) males and 46 (46.9%) females, with 175 teeth evaluated. The mean age of participants was 34.6 ± 11.8 years. Maxillary incisors constituted the largest proportion of the study sample, followed by the mandibular incisors, maxillary premolars, and mandibular premolars. Based on the composite reference standard, 100 (57.1%) teeth were diagnosed with necrotic pulp, whereas 75 (42.9%) were classified with vital pulp (Table 2).

**Table 2:** Baseline characteristics of the study participants and evaluated teeth. Data are presented as mean ± SD for continuous variables and number (percentage) for categorical variables. SD = standard deviation, N = number of participants/teeth.

Characteristic		Value
Total patients (N)		98
Total teeth evaluated (N)		175
Age (years), Mean ± SD		34.6 ± 11.8
Sex, n (%)	Male	52 (53.1%)
	Female	46 (46.9%)
Tooth type, n (%)	Maxillary incisor	78 (44.6%)
	Mandibular incisor	42 (24.0%)
	Maxillary premolar	30 (17.1%)
	Mandibular premolar	25 (14.3%)
Final diagnosis (reference standard), n (%)	Vital pulp	75 (42.9%)
	Necrotic pulp	100 (57.1%)

The diagnostic performance of the individual pulp sensibility tests is presented in Table 3. Among the individual tests, pulse oximetry demonstrated the highest diagnostic accuracy, with an AUC of 0.905, followed by the electric pulp tester (AUC = 0.860), cold test (AUC = 0.825), and heat test (AUC = 0.750). Pulse oximetry also showed the highest sensitivity (93.0%) and specificity (88.0%) among the diagnostic methods.

**Table 3:** Diagnostic accuracy of individual pulp sensibility tests (n = 175). Data are presented as percentage (%) or AUC with 95% CI; diagnostic accuracy was evaluated using ROC curve analysis. AUC = area under the curve, ROC = receiver operating characteristic, CI = confidence interval, PPV = positive predictive value, NPV = negative predictive value.

Test	Sensitivity (%)	Specificity (%)	PPV (%)	NPV (%)	AUC (95% CI)	Youden's Index
Cold test	85.0	80.0	85.0	80.0	0.825 (0.765–0.885)	0.650
Heat test	78.0	72.0	78.8	71.1	0.750 (0.679–0.821)	0.500
Electric pulp tester (EPT)	88.0	84.0	88.0	84.0	0.860 (0.806–0.914)	0.720
Pulse oximetry	93.0	88.0	91.2	90.4	0.905 (0.861–0.949)	0.810

The diagnostic performances of the multimodal models are summarized in Table 4. The parallel model demonstrated the highest sensitivity (97.0%), whereas the serial model showed the highest specificity (97.3%). However, the weighted GEE logistic regression model showed the best overall diagnostic performance with a sensitivity of 91.0%, specificity of 90.7%, and the highest AUC (0.933), indicating superior discrimination between vital and necrotic pulps compared with other multimodal approaches.

**Table 4:** Diagnostic performance of the combined multimodal diagnostic models. Data are presented as percentage (%) or AUC with 95% CI, the weighted model was generated using binary logistic regression, ROC curve analysis was used to determine the optimal cut-off using Youden's index. AUC = area under the curve, ROC = receiver operating characteristic, CI = confidence interval, PPV = positive predictive value, NPV = negative predictive value, GEE = generalized equation estimate.

Model	Interpretation rule	Sensitivity (%)	Specificity (%)	PPV (%)	NPV (%)	AUC (95% CI)	Youden's Index
Parallel model	“OR” rule (any test positive for necrosis)	97.0	65.3	78.9	94.2	0.811 (0.748-0.874)	0.623
Serial model	“AND” rule (all tests positive for necrosis)	68.0	97.3	97.1	69.5	0.825 (0.765-0.885)	0.650
Weighted GEE (Logistic) model	Predicted probability from regression ≥ optimal cut-off	91.0	90.7	92.9	88.3	0.933 (0.896-0.970)	0.817

Pairwise comparisons between the multimodal models and the best-performing individual tests are shown in Table 5. The weighted logistic regression model demonstrated a significantly greater AUC than pulse oximetry (p = 0.032) and significantly outperformed the parallel model (p < 0.001), whereas the parallel and serial models exhibited lower overall diagnostic accuracy, despite their respective advantages in sensitivity and specificity.

**Table 5:** Pairwise comparison of the diagnostic accuracy of the combined models and the best individual test. Data are presented as difference (Δ) in diagnostic performance; DeLong's test was used to compare AUCs and McNemar's test was used to compare paired sensitivity and specificity. AUC = area under the curve, *p < 0.05 were considered statistically significant.

Comparison model pair	Δ Sensitivity	p-value (McNemar)	Δ Specificity	p-value (McNemar)	Δ AUC	p-value (DeLong)
Parallel model vs. Pulse oximetry (Best single test)	+4.0%	0.371	-22.7%	< 0.001*	-0.094	0.041*
Serial model vs. Pulse oximetry (Best single test)	-25.0%	< 0.001*	+9.3%	0.041*	-0.080	0.021*
Weighted (Logistic) model vs. Pulse oximetry (Best single test)	-2.0%	0.652	+2.7%	0.581	+0.028	0.032*
Weighted (Logistic) vs. Parallel model	-6.0%	0.180	+25.4%	< 0.001*	+0.122	< 0.001*

Multivariable logistic regression analysis (Table 6) identified pulse oximetry as the strongest independent predictor of pulp necrosis, followed by the electric pulp tester and cold test, while the heat test did not demonstrate a statistically significant independent association (p = 0.052). The weighted model showed good calibration according to the Hosmer-Lemeshow goodness-of-fit test.

**Table 6:** Multivariable logistic regression model predicting pulp necrosis. Data are presented as coefficient (β), adjusted OR with 95% CI, standard error, Wald χ² statistic, and p-value, binary logistic regression was used for model construction; model calibration was assessed using the Hosmer–Lemeshow goodness-of-fit test. β = regression coefficient, OR = odds ratio, CI = confidence interval, χ² = chi-square statistic, *p < 0.05 were considered statistically significant.

Predictor variable (Index test)	Coefficient (β)	Adjusted odds ratio (95% CI)	Standard error	Wald χ²	p-value
Cold test (Negative)	1.35	3.86 (1.69-8.79)	0.42	10.34	0.001*
Heat test (Negative)	0.68	1.97 (0.99-3.92)	0.35	3.78	0.052
Electric pulp test (No response)	1.82	6.17 (2.41-15.82)	0.48	14.38	< 0.001*
Pulse oximetry (Absent signal)	2.41	11.13 (3.79-32.73)	0.55	19.20	< 0.001*
Constant	-2.85	-	0.51	31.19	< 0.001*

**Figure 1:**
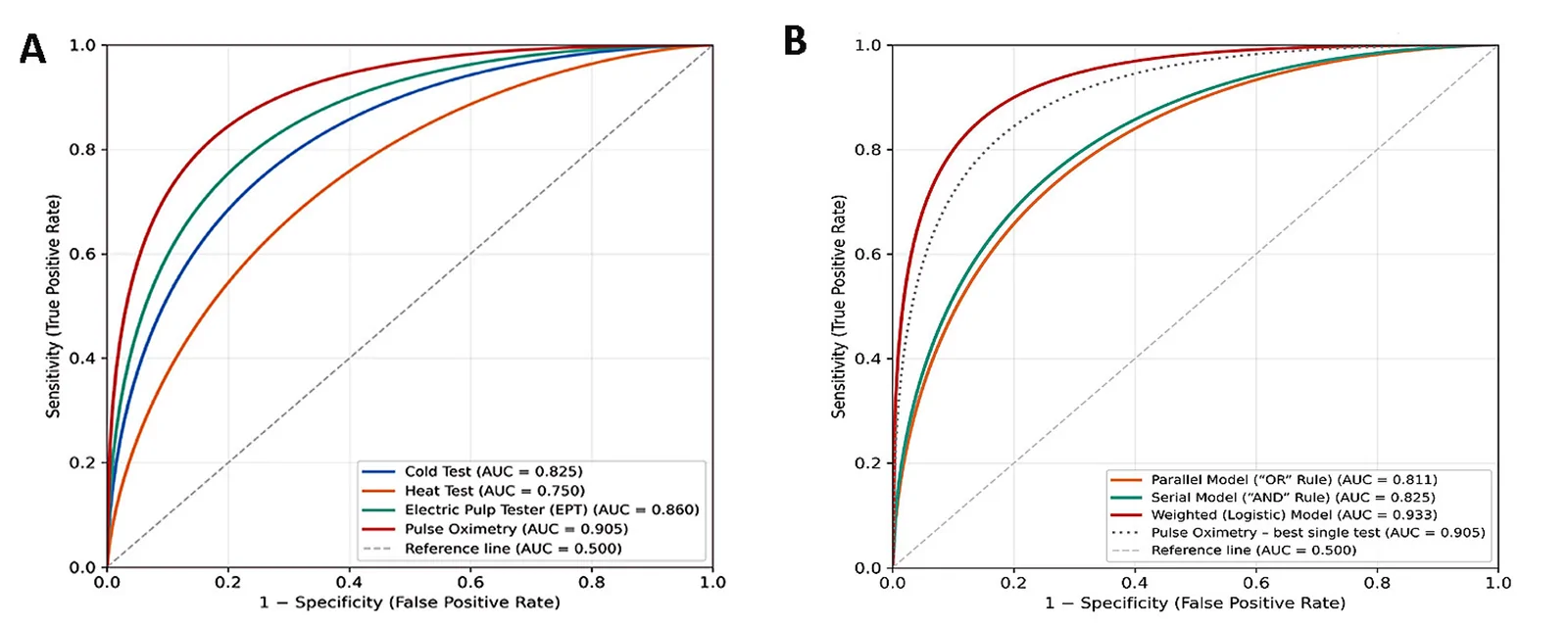
(A) ROC curve for individual pulp sensibility test, (B) ROC curve for combined multimodal diagnostic test. ROC = receiver operating characteristic curve, AUC = area under curve.

The ROC curves for individual pulp sensibility tests are shown in Figure 1A. Pulse oximetry demonstrated the largest area under the ROC curve, followed by the electric pulp tester, cold test, and heat test, confirming its superior diagnostic accuracy among individual pulp sensibility test. Figure 1B shows the ROC curves for the combined multimodal diagnostic models. The weighted logistic regression model exhibited the highest area under the ROC curve (AUC = 0.933) and outperformed both the parallel and serial models, as well as the best individual test (pulse oximetry), indicating that combining multiple pulp sensibility tests using a weighted approach provides the greatest overall diagnostic performance.

## Discussion

Accurate assessment of pulpal health remains one of the most critical aspects of endodontic diagnosis, because treatment decisions depend largely on the correct differentiation between reversible pulpal inflammation, irreversible pulpitis, and pulpal necrosis. Conventional pulp sensibility tests evaluate the neural response of the pulp rather than its vascular supply, making it susceptible to false-positive and false-negative responses under several clinical conditions [1]. This prospective diagnostic accuracy study compared the performance of four commonly used pulp sensibility tests, individually and in combination. The findings demonstrated that pulse oximetry achieved the highest diagnostic accuracy among the individual tests, whereas a weighted multimodal diagnostic model integrating all four tests produced the greatest overall diagnostic performance.

Among individual diagnostic methods, pulse oximetry exhibited the highest sensitivity, specificity, and area under the ROC curve. This finding is biologically plausible because pulse oximetry measures pulpal oxygen saturation, which directly reflects the vascular status of the dental pulp rather than the sensory nerve function. Pulpal blood flow persists despite transient neural dysfunction caused by trauma, orthodontic movement, calcification, or recent restorative procedures; pulse oximetry provides a more objective assessment of vitality than conventional sensibility tests [6]. These findings agree with those reported by Jafarzadeh and Rosenberg [7] and Ghouth et al. [8], who concluded that pulse oximetry consistently demonstrates superior diagnostic accuracy and greater reproducibility than traditional pulp-testing methods. Likewise, Setzer et al. [9] reported that vascular-based assessment is more reliable than neural response in determining true pulp vitality.

The electric pulp tester demonstrated the second-highest diagnostic performance in the present study. An electric pulp tester stimulates sensory nerve fibers within the pulp and generally provides reliable results in mature permanent teeth with intact neural innervation. However, its accuracy may decrease in teeth with calcified canals, recently traumatized teeth, immature apices, or in patients with altered pain perception [1]. The present findings are consistent with the observations of Petersson et al. [10] and Mejàre et al. [11], who reported relatively high sensitivity and specificity of the electric pulp tester, but emphasized that its diagnostic accuracy depends on the integrity of pulpal nerve fibers rather than tissue vitality itself.

Cold testing also demonstrated good diagnostic performance and outperformed the heat test. Cold stimuli induce rapid outward movement of dentinal fluid, activating A-delta nerve fibers located near the odontoblastic layer and producing a prompt sensory response in vital teeth [12]. In contrast, heat testing exhibited the lowest diagnostic accuracy in the present investigation. Heat-induced responses are generally less consistent because they depend on C-fiber activation, which occurs primarily in inflamed pulps and is influenced by the restoration thickness, remaining dentin, and patient pain thresholds. A systematic review and meta-analysis by Mainkar and Kim [13] reported that pulse oximetry demonstrated the highest diagnostic accuracy among commonly used pulp sensibility tests, whereas the heat test showed the lowest accuracy. The present study showed similar findings, with pulse oximetry achieving the highest diagnostic performance among the individual tests, and the heat test exhibiting the poorest performance. These results further support the superiority of vascular-based pulp assessment over conventional neural response tests in evaluating pulpal health. Similar findings were reported by Brignardello-Petersen [14].

An important finding of the present study was the improved performance achieved by multimodal diagnostic models. The parallel model demonstrated the highest sensitivity, indicating its usefulness as a screening strategy in which minimizing false-negative diagnoses is clinically desirable. Because a tooth was classified as necrotic when any individual test suggested necrosis, this approach maximized disease detection, but inevitably reduced specificity by increasing false-positive diagnoses. Conversely, the serial model required agreement among all tests before classifying a tooth as necrotic, resulting in excellent specificity, but considerably lower sensitivity. These findings are consistent with established principles of diagnostic test combinations, where parallel testing increases sensitivity, whereas serial testing improves specificity [15].

The weighted logistic regression model achieved the highest overall diagnostic accuracy with the greatest area under the ROC curve and a balanced combination of sensitivity and specificity. Unlike simple parallel or serial decision rules, logistic regression assigns different statistical weights to each diagnostic test based on its predictive contribution [16]. In the present study, pulse oximetry emerged as the strongest independent predictor of pulpal necrosis, followed by the electric pulp tester and cold test, whereas heat testing contributed little to the final prediction model. These results support the growing application of multivariable predictive models in diagnostic research and highlight the value of integrating complementary diagnostic information rather than relying on a single clinical test [17].

ROC curve analysis further supported these findings. Pulse oximetry demonstrated the highest diagnostic performance among the individual tests, whereas the weighted multimodal model exhibited an even greater area under the curve, indicating superior discrimination between vital and necrotic pulps. This suggests that although pulse oximetry alone performs exceptionally well, incorporating information from additional pulp tests further refines the diagnostic accuracy and reduces diagnostic uncertainty [18].

The findings of this study have several important clinical implications. In routine endodontic practice, clinicians should avoid relying solely on a single pulpal vitality test, particularly in complex clinical situations. Pulse oximetry should be considered as a valuable adjunctive diagnostic tool because it objectively assesses pulpal vascularity and demonstrates the greatest individual diagnostic accuracy. Furthermore, combining multiple diagnostic tests through a structured multimodal approach may substantially improve clinical decision-making, reduce unnecessary endodontic treatment, and facilitate earlier and more accurate diagnosis of pulpal diseases.

The present study had certain limitations. The investigation was conducted at a single institution with a relatively modest sample size, which may have limited the generalizability of the findings. Only anterior teeth and premolars were included, and molars were not evaluated. In addition, the weighted logistic regression model was developed and evaluated within the same study cohort without internal or external validation, and its performance should therefore be confirmed in independent populations before broader clinical application. The specialized dental sensor clip required for pulse oximetry may not be readily available in all routine clinical settings. Future multicenter studies involving larger and more diverse populations, including posterior teeth and different age groups, together with external validation of the predictive model and the development of standardized dental pulse oximetry devices and advanced machine-learning-based predictive models, may further improve the diagnostic accuracy of multimodal pulpal health assessment.

## Conclusions

Among the individual tests evaluated, pulse oximetry demonstrated the highest diagnostic accuracy for assessing pulpal health. Although the parallel and serial multimodal models achieved the highest sensitivity and specificity, respectively, the weighted logistic regression model showed the best overall diagnostic performance by integrating information from multiple pulp sensibility tests. These findings suggest that a multimodal diagnostic approach incorporating pulse oximetry, electric pulp testing, and thermal testing may improve the accuracy of clinical diagnosis and facilitate informed treatment planning in endodontic practice. Further validation in larger, multicenter populations is warranted before broader clinical implementation.
